# Genetic and genomic insights of the Comcáac people

**DOI:** 10.1007/s12687-025-00829-9

**Published:** 2025-09-30

**Authors:** Alejandra Paulina Pérez-González, Israel Aguilar-Ordoñez, Norma A. Caballero, Enrique Morett

**Affiliations:** 1 Computational Systems Biology/Integrative Genomics Laboratory, Instituto Nacional de Medicina Genómica, (INMEGEN), Ciudad de México, 14610 México; 2 Facultad de Estudios Superiores Iztacala, Universidad Nacional Autónoma de México (UNAM), Ciudad de Mexico, 54090 México; 3 Tecnológico de Monterrey, OriGen Project, Monterrey, Nuevo León México; 4 Facultad de Ciencias Biológicas, Benemérita Universidad Autónoma de Puebla, Heroica Puebla de Zaragoza, Puebla 72592 México; 5 Departamento de Ingeniería Celular y Biocatálisis, Instituto de Biotecnología, Universidad Nacional Autónoma de México, Cuernavaca, Morelos 62210 México

**Keywords:** Comcáac, Seri, Genetics, Genomics, Indigenous, Molecular anthropology

## Abstract

The Comcáac (also known as Seri) are an indigenous community from the central coast of the Sonoran Desert in Mexico. Genetic studies conducted on this population since the late 1990s have revealed marked genetic differentiation resulting from pronounced genetic drift caused by a historical bottleneck, which is consistent with anthropological and linguistic records. Research has examined allele frequencies and genetic variants associated with cirrhosis risk, pathogen adaptation, forensic markers, vitamin and sugar metabolism, body mass index, immune responses, cytochrome genes, genetic distances with other groups, and migration patterns. While early investigations relied on classical genetic approaches, more recent studies have employed omics technologies, including whole-genome sequencing. Analyses of the Comcáac genome provide valuable insights into the phenotypic traits and medical predispositions of this community.

**Supplementary Information:**

The online version contains supplementary material available at 10.1007/s12687-025-00829-9.

## Introduction

When we discuss human genetics we refer to molecular characteristics that are intimately intertwined with the cultural, territorial and social identity of communities. Molecular anthropology and population genomics studies unveil aspects of human health and well-being, including genetic diversity, susceptibility to disease, and even adaptations to different environments. The American continent harbors hundreds of indigenous groups. These groups exhibit one of the lowest levels of genetic diversity among continental populations but high divergence among subpopulations (Wang et al. 2007; Moreno-Estrada et al. 2014; Fagundes et al. 2018). As a result, Native Americans harbor unique genetic variants shared among indigenous communities that are rare or even non-existent in other populations of the world (Aguilar-Ordoñez et al. 2021), including functional and medically relevant variants (Moreno-Estrada et al. 2014).

**Fig. 1:**
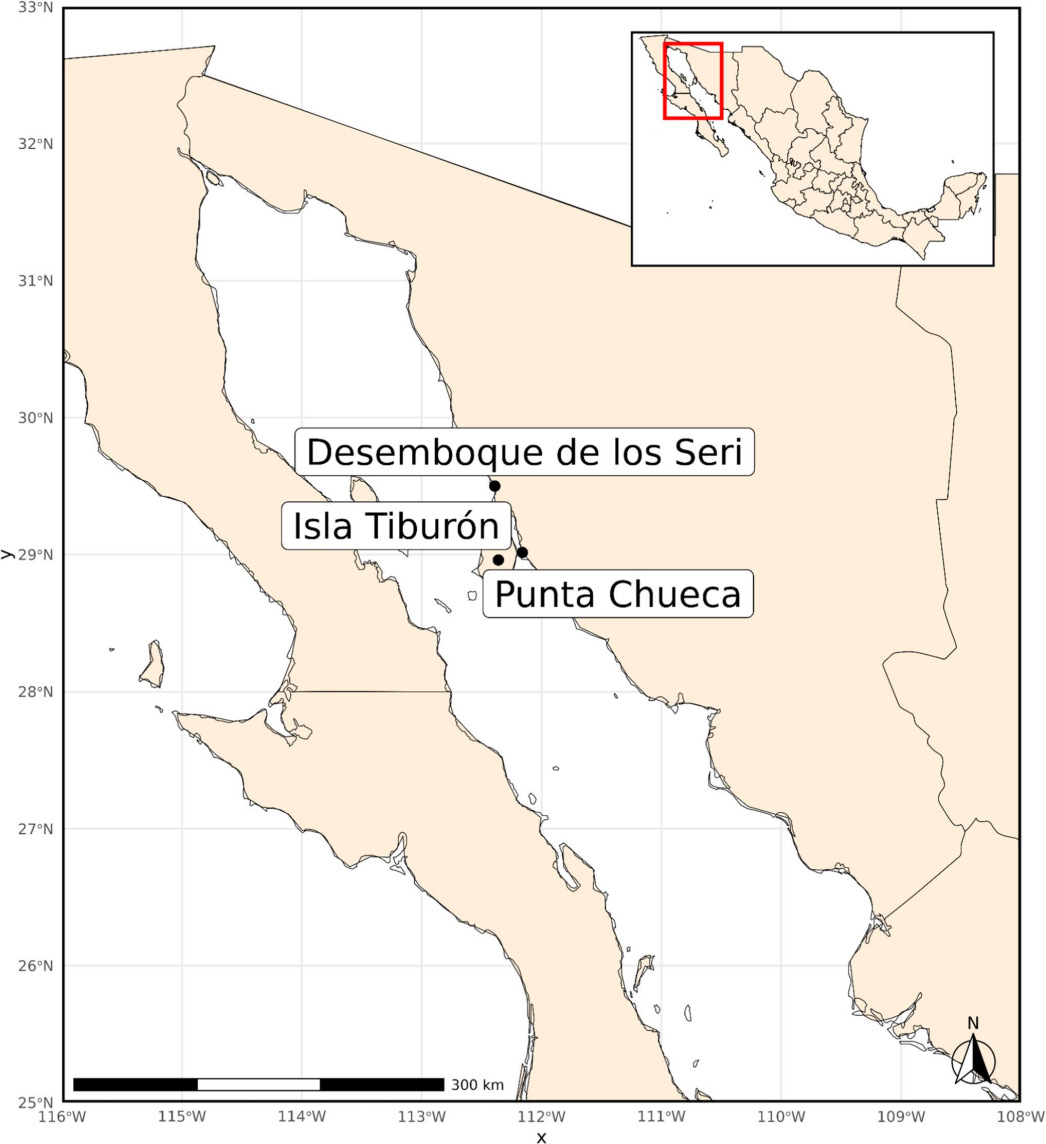
The Comcáac community inhabits two main towns along the coast of Sonora, Mexico, the *Socaaix* (in spanish, Punta Chueca) and *Haxöl ihom* (in spanish, Desemboque de los Seri) communities. Their ancestral territory extends through various semi-permanent settlements along the coastal strip. In addition, they occasionally inhabit islands, such as *Tahejöc* (in Spanish, Isla Tiburón), a place of special cultural and religious significance for them

In recent decades, there have been significant advances in the study of the genetic structure of these populations, which has allowed us to obtain a better understanding not only of the past, but also of the present of the indigenous and cosmopolitan populations of Mexico and the American continent. Within this context, the Comcáac (also known as Seri, Konkaak, or Comcaa’c) are an indigenous community inhabiting *Haant Comcáac*, “the Comcáac Nation”, located in the central part of the Sonoran desert coast, Mexico. According to archaeological evidence, human presence in the ancestral territory of the Comcáac dates back to the Paleo-Indian (Clovis) period, which occurred between approximately 9,000 and 8,700 cal BCE (Ortiz et al. 1972; Bowen 1976; Waters and Stafford 2007). The Comcáac are currently distributed in two populations, called *Socaaix* (in Spanish “Punta Chueca”) and *Haxöl ihom* (“Desemboque de los Seris”) (Fig. 1). The Comcáac are a classically semi-nomadic non-agricultural group with hunter-gatherer traditions (Huitrón 2019).

Due to the arrival of European colonizers, the Comcáac population experienced a significant decrease in size. Initially, the Comcáac were of little interest to the colonial forces, as their territory and lifestyle were not conducive to agriculture or mining. However, the Comcáac endured numerous attempts at extermination, first sanctioned by the Spanish Crown and later continued by the emerging Mexican state in the 19th century (Elizondo and Galván 1999; Luque and Doode 2007). At the beginning of the 20th century, only about 130 Comcáac remained as refugees on Tiburon Island (Sheridan 1999). Later, with the onset of the Mexican Revolution, relations with the Mexican State began to improve, which resulted in an increase in the population (Luque and Doode 2007). In recent years they have experienced a cultural transition as a result of globalization policies in Mexico. This transition has modified their diet and has promoted sedentary habits (Moreno 2013; Lavandera-Torres et al. 2017; Torres 2022). Diseases and risk factors such as obesity (Costa-Urrutia et al. 2020), diabetes, insulin resistance (Ordaz et al. 2015; Robles-Ordaz et al. 2018; Aguilar et al. 2019), high blood pressure, anemia, tuberculosis, kidney, rheumatic and respiratory disease, cancer, gastrointestinal ailments (Narchi et al. 2015), infections (Hector et al. 1996) among others have been reported. Traditionally, they have relied on a complex system of traditional indigenous healing practices, but this has increasingly been replaced by modern healthcare due to factors such as cultural shifts, reduced territorial accessibility (Luque Agraz and Robles Torres 2006; Narchi et al. 2015), and other factors such as the introduction of government health programs. Despite these changes, in recent years the Comcáac population has shown a constant growth at both localities in which they reside (INEGI 2020).

The early history of the Comcáac, characterized by migrations, a drastic reduction of population, and a subsequent rebound in the last 50 years, has left its mark on the genetic makeup we observe today (Pérez González 2022). Compared to the abundance of population genetic studies in other human groups, research on the Comcáac genome is scarce and recent. Indigenous Mexican groups such as the Comcáac may retain genetic variants that were shaped by natural selection in response to their distinct pre-Columbian environments. Compared with more admixed populations, these retained variants, many related to immune function and metabolism, that may contribute to distinct biological characteristics of these groups (Ojeda-Granados et al. 2022; Miron-Toruno et al. 2025).

**Fig. 2:**
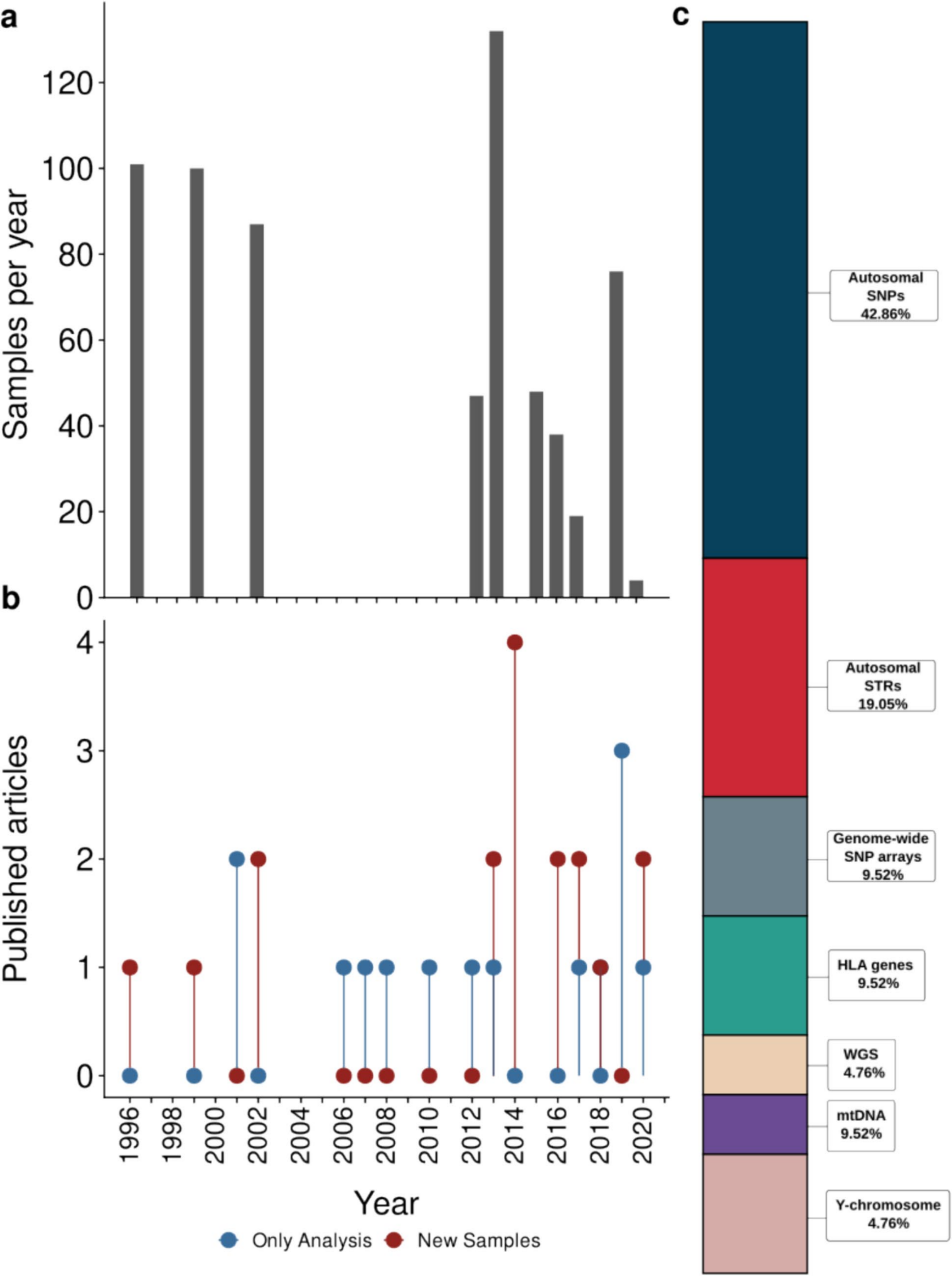
**a** Number of samples published per year. The year with the highest number of sampling reports was 2014. **b** Annual number of articles that collected samples (red) or analyzed samples from previous studies (blue). On average, 0.6 articles are published per year. **c** Percentage distribution of articles reporting new samples in the reviewed literature by type of genetic marker analyzed

It is known that the genetic composition of indigenous populations in Mexico is strongly influenced by geographic barriers and demographic events over time (García-Ortiz et al. 2021), and the Comcáac are a vivid example. However, no comprehensive review has yet examined their historical-demographic and genetic background. Through the lens of molecular anthropology, this article aims to synthesize existing molecular studies to better understand the genetic structure of the Comcáac community, offering an overview of their ancestry and historical origins. Figure 2a presents the annual number of published articles that included or analyzed community samples, along with the corresponding sample counts and the types of genetic markers analyzed.

## The Comcáac genetics

### First genotyping and major histocompatibility complex analysis

The first academic publication examining the molecular genetic aspects of the Comcáac community was published in 1996 (Fig. 2). It reported that the Comcáac are primarily affected by infectious diseases, hypertension, rheumatic conditions, and Type II Diabetes (T2DM). In this study, the major histocompatibility complex II (MHC II) genetic profile of 101 members of 26 families was analyzed. Family segregation analysis showed the presence of only seven haplotypes: DRB10407-DQA103011-OOB10302 (90%); DRB10802-DQA1-0401-OOB10402 (73%); and DRB11402-DQA10501-DQB1*0301 (27%). One DRB1*0404 haplotype was present, along with seven *0701, one *1101, and one *0101 haplotypes, which were reported of Admixed or Caucasian origin. This work suggests for the first time that a bottleneck effect may be responsible for the observed reduction in genetic diversity. Here, the Comcáac community is described as having a genetic profile very similar to that of the natives of the western United States, but different from that of the Mexican Maya and other indigenous populations of South America (Hector et al. 1996).

Later, Infante et al. (1999) reanalyzed the HLA region, genotyping 100 Comcáac belonging to 9 families. Only five HLA-A alleles and seven HLA-B alleles were found. A0201, A68, A31, A24, B3501, B40, B51, B3512, and B15 were present in more than 5% of individuals. B27052 was detected in 2%. A notable finding was the presence of the B27 allele in four individuals, an allele not previously recorded in any other Mexican indigenous community. However, this allele had been documented in Admixed populations (Gorodezky 1992; De Leo et al. 1997). The presence of the B27 allele could be explained by two non-exclusive hypotheses: The first is that this allele could have been introduced into the population due to a founder effect, originated by different migratory waves to the south. The second is that the B*27 allele may have been present in the community as a result of traces of genetic admixture with non-indigenous populations. The B locus was more diverse and the prevalent haplotypes were A*0201-B*3501, A*0201-B*40, A*0201-B*3512, A*31-B*51, A*68-B*3501 and A*68-B*40. This genetic profile was found to be different from the pattern of other Mexican populations. An interesting fact of this work was that it estimated the Comcáac population at approximately 619 individuals, which is valuable because at the time there was no demographic registry of the population. Later, the information from this work was used as a reference for comparison in other studies of HLA regions in Mexican populations (Gorodezky et al. 2001). In addition, data from the study was used to analyze the genetic relationship between Asians and Native Americans through HLA haplotypes. In the latter work, the Comcáac were the only Mexican indigenous group used for comparison. Here, it was determined that Native Americans have a higher genetic affinity with Northeast Asians compared to other major populations in the world (Tokunaga et al. 2001), in agreement with the view that the Comcáac, like other American populations, share a common origin with groups from East Asia and Northeast America.

Balladares et al., (2002) further examined the MHC region, specifically analyzing the TAP1 and TAP2 genes located within the MHC class II region of an interferon-inducible gene cluster. The study included 32 unrelated Comcáac individuals from Desemboque de los Seris and Punta Chueca, along with 89 mestizo residents of Mexico City. The Comcáac individuals included in the study were previously typed for class I and class II genes (Infante et al. 1999). Extended haplotypes were constructed, in which forty-eight haplotypes were defined. Only four QBP-DQB1-QAP-DQA1-DRB1 haplotype variants were found in Comcáac, but when class I data (A and B genes) were added, 23 different haplotypes were found. Finally, after extending the analysis with the TAP1/TAP2 genes, 32 haplotypes were obtained. By analyzing extended haplotypes that included TAP1, TAP2, and multiple MHC class I and II loci, the authors observed that these genes were contributors to the genetic diversity observed in the studied populations. In the same year Alaez et al. (2002) used the same samples to analyze alleles of the DRB1, DQA1 and DQB1 genes and the promoter regions of the DQA1 and DQB1 genes in 31 unrelated, and 24 related Comcáac individuals. Class II genotypes in this population were found to be in Hardy–Weinberg equilibrium, indicating no significant evolutionary forces acting on these genes (Hartl and Clark 2007). Allele frequencies were calculated and haplotypes of these genes were identified. Genotyping data from the previously generated HLA region were also used to make comparisons with other native groups such as the Teneek (Vargas-Alarcón et al. 2006), Nahuas (Vargas-Alarcon et al. 2007), Mayans (Barquera et al. 2020), and with other American, South American, Asian, and Pacific populations (Arnaiz-Villena et al. 2010; Rey et al. 2012; Zúñiga et al. 2013).

### Genetic forensic markers characterization

Historically, STR forensic databases, such as the Combined DNA Index System (CoDIS), lacked sufficient genetic data from Native American populations. This limited representation posed challenges for forensic identification and the accurate interpretation of DNA profiles in paternity testing. To address this issue, genetic profiles of various Indigenous groups were incorporated to ensure their proper representation in these databases. 15 Short Tandem Repeats (STR) loci were typed in eight Native American populations. Among them, 28 Comcáac individuals were analyzed. In addition, genetic distances and pairwise comparisons between populations were estimated from the STR genotyping. Populations from northwestern Mexico (Tepehuanos, Huicholes, Mexicaneros and Coras) showed a greater genetic similarity to each other compared to native groups from the north (Tarahumaras, Mayos, Comcáac and Guarijío) (Rangel-Villalobos et al. 2013). Later 29 Comcáac individuals were typed at Globalfiler® STR markers (i.e. 13 CODIS loci to date, which are CSF1PO, FGA, TH01, TPOX, VWA, D3S1358, D5S818, D7S820, D8S1179, D13S317, D16S539, D18S51, D21S11), and eight additional autosomal STR loci (D1S1656, D2S441, D2S1338, D10S1248, D12S391, D19S433, D22S1045, and SE33) (Ng et al. 2016). The Comcáac exhibited six unique allelic variants that were not found in any other population studied. This discovery aligns with the number of private variants identified in other indigenous populations included in the study. Derived from the typing of these loci, an index of genetic differentiation FST (Wright’s fixation index FST) (Bhatia et al. 2013) was calculated by McCulloh et al. (2016). The Coras, Huicholes and Comcáac were the only Mexican native populations studied here. The FST index was 0.052 between the Comcáac and the Cora, and 0.068 between the Comcáac and the Huichol, indicating a moderate degree of genetic divergence among these populations. The Coras and Huichols showed the least differentiation (0.019) of the Mexican populations, suggesting a greater genetic similarity between them. The observed FST values of the Comcáac could be explained by limited gene flow due to their physical isolation from other populations. Other studies have utilized STR data from Comcáac individuals for forensic purposes, identifying them as a highly genetically differentiated population compared to other Native Mexican groups (Martínez-Cortés et al. 2019).

### Tracing the past with molecular fingerprints

Over the past century, multiple hypotheses have been proposed to explain the major migrations from Asia that populated the Americas. A broad consensus now holds that Native American populations descend from a founding group originating in Northeast Asia that occupied Beringia during the Last Glacial Maximum, subsequently dispersing southward into the continent. Mitochondrial DNA analyses indicate an early single migration followed by diversification within the continent, whereas genome-wide data reveal multiple streams of Eurasian gene flow, and the rapid divergence of South American lineages shaped by isolation and drift (Bonatto and Salzano 1997; Skoglund et al. 2015; Moreno-Mayar et al. 2018; Gusareva et al. 2025). Although genetic data alone cannot determine the precise number of migrations, the discovery of a unique autosomal variant absent in Asia yet ubiquitous across the Americas offers an important marker for testing the hypothesis of a single founding population. In 2003, Zhivotovsky et al. analyzed genotypes from the HGDP-CEPH human genome diversity panel (HGDP) for 377 microsatellites. A 275 bp allele at D9S1120 (also known as GATA81C04 or GATA11E11) was found with high frequency in all American populations in the study (Pima, Mayan, Colombian, Karitian and Surui) and absent in 47 other populations worldwide. This allele was a 9-repeat microsatellite at D9S1120, also known as D9S1120 9RA. The lack of regionally specific private alleles with a high frequency, the characteristic distribution of 9RA, and the rarity of intermediate-sized alleles strongly suggested that all or nearly all copies of 9RA derived from a single mutational event. This allele had a frequency of 36.5% in the pooled American sample, while no other allele was private to a major geographical region (sub-Saharan Africa, Europe Asia south and west of the Himalayas, North Africa, East Asia, Oceania and the Americas) with a frequency above 13%. Expansion of the dataset to 783 loci and 9346 alleles (Rosenberg et al. 2005) did not reveal any additional regionally private allele. To determine whether the D9S1120 9RA allele is specific to American populations, Schroeder et al. (2007) sampled seven populations in the Altai region of eastern Central Asia and eastern Siberia, two Aleutian-Eskimo populations, two Na-Dene populations, and nine Native North American populations, including Cherokee, Chippewa, Huichol, Mixtec, Northern Paiute, Sioux, Jemez, Creek, Karitiana, Surui, Maya, Pima, and 15 Comcáac individuals. D9S1120 9RA was present in all sampled North and South American populations at an average frequency of 32.9%. The allele was also observed in the Koryaks and the Chukchi of Western Beringia but was not found in putative Asian ancestral populations. The Comcáac people exhibited an outlier status with the lowest frequency of the D9S1120 9RA allele at ~ 10.0%. Aguilar-Velázquez et al. (2017) evaluates how the inclusion of D9S1120 9RA allele in population analysis improves mixture estimates and the resolution of population relationships. Here it is reported that the Comcáac group exhibited relatively low allelic diversity at the D9S1120 locus, with only five alleles present out of the nine observed across all populations. Multidimensional scaling (MDS) and pairwise FST analyses based on this locus showed that the Comcáac clustered with other northern and western Mexican Native and Admixed populations, while maintaining distinctiveness relative to certain groups. In both Neighbor-Joining (NJ) and STRUCTURE analyses, the Comcáac contributed to regional clustering patterns of northern and western Mexican populations. For instance, in the STRUCTURE analysis, the Huichol genetic component appeared partially in nearby groups such as the Tepehuano, Mexicanero, and Cora, whereas the Comcáac retained a distinct allele composition, reflecting both geographic and genetic differentiation. This can be attributed to their identification as potential isolates, as suggested by linguistic data (Moser and Marlett 2010).

Another important aspect with the potential to shed light on migration patterns is the study of mtDNA and Y-chromosomes. Both mtDNA and Y chromosome diversity can be used to investigate proposed migration events. In North America, the frequencies of mtDNA haplogroups show regional continuity, offering insights into the relationships among populations in these areas (Peñaloza-Espinosa et al. 2007). To understand the genetic structure of the descendants of the Hohokam and Anasazi cultures, as well as the Uto-Aztecan and Southern Athapaskan migrations into or out of the Southwest region, the mitochondrial haplogroups of a total of 117 Native Americans were determined by restriction fragment length polymorphism (RFLP). Also, a subset of 53 samples was sequenced from nucleotide positions (np) 16055–16548. The study included eight Comcáac individuals. Lineage B is the predominant haplogroup in the Southwestern United States and northwestern Mexico. Most populations in the Aridoamerica region exhibit high frequencies of haplogroups B and C. The Comcáac population, in particular, shows frequencies of 0.125 for haplogroup B and 0.875 for haplogroup C (See Table 1). The most common B haplotype, shared by the Navajo, Zuni, Jemez, and Comcáac, is characterized by mutations at np 16111 and np 16483. The founding haplotype for haplogroup C is a key genetic variant that serves as a reference point for the lineage of haplogroup C (Zhong et al. 2010). This founding haplotype is found in both the Shoshone people and certain central Uto-Aztecan groups. Additionally, it is present in three Comcáac individuals. This may indicate that these groups share a common genetic ancestor. Despite the shared founding haplotype, the network reveals that the three Uto-Aztecan groups (excluding the Shoshone and central Uto-Aztecan groups) have distant genetic relationships, suggesting at the same time significant genetic divergence among these groups. The Comcáac individuals who do not share the founding haplotype are clustered together. They all have a specific genetic mutation at nucleotide position np 16301, which indicates a close genetic relationship among them. Haplogroup C fixation in the Comcáac was unusual (Malhi et al. 2003), reflecting intense genetic drift in this very small and isolated population (Infante et al. 1999). mtDNA haplotype networks for haplogroups A, B, and C based on HVSI sequences were constructed by Malhi et al. (2003).

**Table 1:** Summary of mtDNA and Y chromosome haplotype frequencies reported for Comcáac individuals. The mtDNA data show a predominance of haplogroup C in both bibliographic sources (Malhi et al. 2003; McCulloh et al. 2016), while the Y-chromosome data reported by Malhi et al. (2008) indicate the presence of haplogroup Q with the absence of haplogroup C and R. Proportions are shown in percentages

Type	N	Haplogroup A	Haplogroup B	Haplogroup C	Haplogroup D	Haplogroup X	Reference
mtDNA haplogroups	8	0%	12.5%	87.5%	0%	0%	(Malhi et al. 2003)
	29	0%	13%	86%	0%	0%	(McCulloh et al. 2016)
Y chromosome haplogroups	**N**	**Haplogroup Q**	**Haplogroup C**	**Haplogroup R**	**-**	-	**Reference**
	15	100%	0%	0%	-	-	(Malhi et al. 2008)

To test migration hypotheses, several genetic markers have been analyzed in Indigenous populations. Malhi et al. (2008) conducted a study on Y chromosome variation among Athapaskan-speakers to gain insights into the population history of males within this group. Specifically, they examined whether Athapaskans in the Subarctic and Southwest share Y chromosomes, to the exclusion of non-Athapaskans in the Southwest, indicating recent common ancestry. To test similarities and differences, the membership in Y chromosome haplogroup Q, C, or R was determined for 231 males from 12 populations in this study, including 15 Dogrib, 23 San Carlos Apache, 13 Jemez, 38 Akimal O'ohdam, 13 Tohono O'ohdam, 20 Tarahumara, 37 Cora, 18 Huichol, 7 Nahua of San Pedro Atocpan, 10 Nahua of Cuetzalan, 22 Mixtec, and 15 Comcáac. Haplotypes were determined for eight STRs: DYS19, DYS390, DYS391, DYS392, DYS393, DYS389I, DYS389II, and DYS439. The Comcáac population exhibited a null frequency for haplogroups C and R, while haplogroups N and Q were present in the same proportion. However, specific frequencies for these Y chromosome haplogroups were not clearly delineated for Comcáac.

A more recent study explored if droughts, as evidenced by archaeological clues, shifted southward the boundary between Arid America and the green and culturally rich Mesoamerica (Villa-Islas et al. 2023). The latter region was home to civilizations such as the Aztecs and the Maya. The hypothesis suggests that this climate change led to the replacement of the population on the northern border of Mesoamerica by semi-nomadic hunter-gatherers from Aridoamerica. However, this hypothesis was based solely on archaeological data. To test it, ancient genomic and mitochondrial DNA data were generated. These data allowed not only to evaluate the drought hypothesis, but also to describe the ancient population structure of Mexico and to investigate the contribution of unsampled genetic lineages to the ancient genomes. The study included genetic information from the Comcáac population, previously characterized by DNA arrays (Moreno-Estrada et al. 2014), taking them as the representative native population of northern Mexico for the ADMIXTURE model. In this model, the Comcáac were grouped, as in past studies, in a single cluster without admixture with the other populations within the analysis. Genetic continuity was found in ancient individuals from before and after the climate change episode. This contradicts the hypothesis of population replacement by Arido-American groups in this region and suggests that the local population remained in its place of origin despite the prolonged droughts (Villa-Islas et al. 2023).

An interdisciplinary approach combining linguistic, archaeological, and genetic data offers potential for elucidating processes such as long-distance migration, gene flow, and cultural diffusion that likely shaped the contemporary genetic distribution among the Comcáac. While investigations at the mitochondrial haplotype and Y chromosome levels have yielded valuable insights, definitive conclusions regarding specific Comcáac migration patterns remain elusive. A meta-analysis centered on haplotype data for this population could provide a promising avenue for further exploration. A summary of the haplogroups reported for Comcáac individuals is shown in Table 1.

In 2014 ~ 475,109 SNPs were analyzed using array technology in 104 individuals from seven Indigenous populations: six from the Pacific Northwest and one from Mexico, the Comcáac. These data were subsequently integrated with results from a previous investigation. The aim of the study was to understand the genetic relationship of Pacific Northwest populations relative to other populations, both on a global and continental scale. Genome-wide mean haplotype heterozygosity was 0.615 (SD = 0.115) across the Comcáac, Pima, and Maya populations. Results of ADMIXTURE, MDS and haplotype heterozygosity analyses showed differences in genetic structure between Pacific Northwest populations and those of Central and South America, reflecting both pre-contact divergence and post-contact admixture histories and providing a comparative framework for understanding the effects of European colonization on indigenous communities across the continent (Verdu et al. 2014).

Using DNA arrays, genomic variation within Mexico was studied in over 1,000 individuals representing 20 indigenous and 11 Admixed populations. The study found a striking genetic stratification among indigenous populations within Mexico at varying degrees of geographic isolation. The Comcáac and Lacandon groups in Mexico were shown to be the communities with the highest genetic differentiation from each other (FST = 0.136). This value is even higher than the FST between European and Chinese populations in the HapMap3 project, indicating remarkable genetic diversity among communities (Moreno-Estrada et al. 2014). Like other Amerindian communities, the Comcáac present a high rate of autozygosity, evident in the presence of long runs of homozygosity (ROH) in their genome. Notably, more than 10% of the Comcáac genome consists of ROH, a proportion significantly higher than that found in other native populations. The relatively small population size enhances the effects of genetic drift, contributing to the high FST values observed. In the PCA (Principal Component Analysis) and ADMIXTURE analyses, the Comcáac form a distinct cluster, highlighting them as one of the three most isolated and genetically restricted communities, along with the Lacandones and Tojolabales. The findings of this study suggest that the genetic makeup of present-day Mexicans reflects the ancient substructure of Native American populations. This substructure has been maintained despite the possible homogenizing effect of mestizaje that occurred after colonization.

Until 2021, genetic studies of the Comcáac population primarily utilized traditional molecular biology techniques and DNA array methods. A comprehensive study involving high-coverage whole-genome sequencing of 76 unrelated individuals from 27 Indigenous groups across Mexico was reported, including two Comcáac women and two men. This study, utilizing whole genome sequencing, revealed that the Comcáac exhibited the greatest genetic divergence among the groups studied. In PCA and ADMIXTURE analyses, the Comcáac group was distinctly separated as an isolated lineage. They presented the highest number of novel variants (2,496 unique SNVs, 8 of which were fixed), the lowest number of singletons among the studied populations, and the most specific genotypes (Aguilar-Ordoñez et al. 2021), supporting the evidence of an unique genomic context that remains largely unexplored.

### Evidence of genetic selection pressures

Positive selection acting on genes and genomes can elucidate the evolutionary reasons behind variations observed between species and among different populations within a species (Wagner 2007). Ojeda-Granados et al. (2022) conducted selection scans using the nSL method on the Comcáac population and identified three gene networks comprising a total of 10 genes. These include SI, HK2, and PGM1, which are involved in starch and sucrose metabolism; RASGRP3, CACNA1E, CACNA1D, and CACNA2D1, which participate in MAPK signaling; and GNG2, PRKACB, and CALM1, associated with Ras signaling. These genes display strong signatures of positive selection in the Comcáac population. Genes SI, HK2, PGM1 also play roles in the regulatory steps of glycolysis/gluconeogenesis and the insulin signaling pathways. These loci are also reported in the KEGG database as involved in the pathogenesis of some metabolic disorders, among which T2DM (Muller et al. 2007). The selective events in these genes appear to be consistent with the Comcáac diet and lifestyle, which has long depended on the consumption of succulent fruits like *Óol* (in spanish, pitaya, *Stenocereus thurberi*) that are rich in simple sugars (Palma and Cassiano 2003). Therefore, the authors speculate that selective events involving loci contributing to the metabolism of sugars, as well as to signal transduction initiated by sweet food intake, might have optimized the utilization of the high amounts of ingested sugars as energy sources or reserves. This could also reduce the potential metabolic risk associated with glucose overload. It is unclear what specific pressures could have triggered such adaptive events, although it is noteworthy that some of these genes play a role in the sweet taste signaling pathway and functional cascade leading to insulin secretion. The other two significant genetic networks reported included seven genes involved in the Ras and MAPK signaling pathways, which regulate basic functions such as cell proliferation and differentiation (Morrison 2012). It remains unclear which specific selective pressures might have triggered such adaptive events. Some of these genes, such as CACNA1E and CACNA1D, are also expressed in pancreatic beta cells and play roles in both the sweet taste signaling pathway and the functional cascade leading to insulin secretion (Vajna et al. 1998). Interestingly, these loci have also been implicated in the pathogenesis of T2DM (Muller et al. 2007). Also, a comparative analysis of genetic adaptation using WGS data identified nonsynonymous variants putatively under selection within a northern Mexican Indigenous cluster, which included the Comcáac. Analysis revealed nonsynonymous variants under putative positive selection in key immune-related genes such as ID3, GBP1, XIRP1, and MUC19, all implicated in host defense against diverse pathogens. Notably, GBP1 and XIRP1 are involved in immune responses to *Salmonella spp.*, (Fisch et al. 2019). The presence of selection signals in MUC19, previously linked to resistance against parasitic and viral infections (Hicks et al. 2000) and carrying a Denisovan-like haplotype in admixed Latin Americans, emphasizes the role of pathogen-driven adaptation in Comcáac evolutionary history. However, the authors caution that the high genetic drift and reduced diversity observed in the Comcáac, likely due to a small long-term effective population size and extensive runs of homozygosity, necessitate careful interpretation of these region-specific results, as drift may have contributed to the observed patterns of allele frequency change alongside pathogen-mediated selection (Miron-Toruno et al. 2025).

## Genetic characteristics of biomedical interest in the Comcáac people

### Cytochrome enzymes in the Comcáac people

Discussions about precision medicine have predominantly centered on population differences in drug pharmacokinetics and pharmacodynamics within developed countries. However, for precision medicine to be truly global, it is essential to characterize drug metabolism and drug targets across diverse populations, including indigenous societies (de Andrés et al. 2017). Certain regions of the genome crucial for human health have been investigated in the Comcáac population, including cytochromes involved in xenobiotic metabolism (see Table 2). CYP genes are highly polymorphic and contribute significantly to interindividual and interethnic differences in drug metabolism in humans (Gardiner and Begg 2006).

**Table 2:** Allele frequencies of the CYP2C9 and CYP2D6 allelic and genotypic frequencies reported in various studies on the Comcáac people. It also includes information on the drugs metabolized by these enzymes and the corresponding references

Cytochrome	Allele frequency	Metabolized drugs	Reference
CYP2C9*1	97.4% (0.86–0.99 95% CI)	Fluoxetine, losartan, phenytoin, tolbutamide, torsemide, S-warfarin, and numerous Non-steroidal anti-inflammatory drugs (NSAIDs).	Sosa-Macías et al. (2013) (*n* = 19)
CYP2C9*2	2.6% (0.00–0.1495% CI)		
CYP2D6*1	69.7%		
CYP2D6*2	5%	Antidepressants, antipsychotics, antiarrhythmics, antiemetics, b-adrenoceptor antagonists (b-blockers) and opioids.	Lazalde-Ramos et al. (2014) (*n* = 19)
CYP2D6*4	21%		
CYP2D6 Multifunctional (duplications)	5.2%		

The CYP2C9 enzyme metabolizes drugs across many therapeutic classes including fluoxetine, losartan, phenytoin, tolbutamide, torsemide, S-warfarin, and numerous Non-steroidal anti-inflammatory drugs (NSAIDs) (Vélez Gómez et al. 2018; Alvarado et al. 2019). In 2004, Schurr anticipated that CYP2C9 variant allele frequencies among Native Americans populations might parallel those observed in some Asian groups, given their shared ancestry. This perspective provided the basis for the hypothesis tested by Sosa-Macías et al. (2013) who investigated the distribution of CYP2C9*2, *3, and *6 in eight Indigenous groups from northwestern Mexico, comparing their results with published frequencies from Asian and other Native American populations. In the Comcáac, 19 individuals were genotyped at this locus. The wild-type CYP2C91 allele was the most frequent (97%), while CYP2C92 was detected at low frequencies, appearing only in the Comcáac (AF = 2.6%) and Mayan (AF = 5.7%) populations. CYP2C9 is involved in the metabolism of approximately 15–20% of clinically used drugs (Zanger and Schwab 2013; Bao et al. 2025). CYP2C9*2 allele is linked to a change in amino acid (Arg144 Cys144) that produces a reduction in enzyme activity in genomes of European populations (Fekete et al. 2021). Only one subject in the Comcáac group (5.26%) with the heterozygote status CYP2C9*1/*2 was identified. However, no homozygote *2/*2 neither the CYP2C9*3 and CYP2C9*6 alleles were detected in the Comcáac. Also, 11% of European ancestry was observed in the Comcáac population. These findings could explain the unexpected presence of the CYP2C9*2 allele in the Comcáac group, as this allele is more prevalent in European and Euro-American populations (Scordo et al. 2004; Fekete et al. 2021).

Another cytochrome analysis typed polymorphisms in the CYP2D6 gene. The CYP2D6 gene is a member of the CYP450 family and is involved in the metabolism of many widely used drugs, including antidepressants, antipsychotics, antiarrhythmics, antiemetics, b-adrenoceptor antagonists (b-blockers), and opioids (Teh and Bertilsson 2012). CYP2D6 is highly polymorphic, with more than 100 allelic variants identified to date. Some of these alleles are associated with increased, decreased or absent enzymatic activity (Zhou 2009). The CYP2D6*4 allele is clinically important and often causes alterations in drug response. Of the CYP2D6 alleles, *1, *2, *35, *1xN, and *2xN demonstrate normal or increased activity, while *10, *17, *29, and *41 show reduced activity, while alleles *3, *4, *5, and *6 are inactive (Llerena et al. 2007; Kane 2025). The study determined the allelic distribution of CYP2D6 in eight Indigenous groups from northwestern Mexico, including 19 Comcáac individuals, and compared the frequencies in Mexican admixed individuals. The Comcáac group had the highest frequency of the non-functional CYP2D6*4 allele (21.05%), and the lowest allelic diversity (absence of *5, *6, *10, *41, *3, *17, *35, *29), as well as a notable percentage of intermediate (12.01%) and ultra-rapid (8.64%) metabolisers, with the majority being extensive metabolisers (75.2%) (Lazalde-Ramos et al. 2014). CYP2D6*4 allele is clinically significant, frequently leading to altered drug clearance and response (Zhou 2009; Lyon et al. 2012). The high frequency of intermediate metabolizer (IM) among the Comcáac could make them more susceptible to presenting adverse events when they are medicated with CYP2D6 substrates, as has been reported in psychiatric patients with the PM or IM phenotype (Milosavljević et al. 2021; Molden and Jukić 2021). Despite its association with impaired metabolization in these populations, the influence of the CYP2D64 allele in the Comcáac population has not yet been deeply studied.

The CYP1A2, CYP2C9, CYP2C19, CYP2D6, and CYP3A4 genes were also phenotyped and genotyped in 10 Mexican indigenous individuals, including 10 members of the Comcaac’c community. Genotyping was accompanied by phenotyping for the drugs omeprazole (20 mg), losartan (25 mg), and caffeine (100 mg) followed by dextromethorphan (30 mg) one hour after administration. The ratio of losartan to losartan carboxylic acid was significantly greater in CYP2C9*2 or *3 homozygotes or carriers, compared to those with the reference genotype. The minor allele frequencies (MAFs) of the CYP2C19*2, *3, and *17 alleles were found to be 12.0%, 0.2%, and 2.2%, respectively, among individuals from the Tarahumara, Tepehuano, Mexicanera, Huichol, Cora, Mayo, Guarijío, and Comcáac populations. These frequencies represent the combined data from all eight populations. The findings indicate that current personalized medicine strategies, which predict phenotypes based on genotypes of alleles common in European populations, are not applicable to admixed individuals and Native American populations (de Andrés et al. 2017). This and other studies demonstrate the genetic variability of the Native Mexican population and highlight the importance of studying each population separately. By analyzing each cytochrome in Native North American populations, the unique genetic profiles of these groups can be better understood (Henderson et al. 2018). Genotyping is useful; however, it does not account for all metabolic phenotypes. Further research on the metabolic phenotypes of the main CYP enzymes is needed for an accurate prediction of individuals drug responses.

### Areas of metabolic interest in the Comcáac people

The study of genetics in the Comcáac people is not limited only to the frequency of cytochromes and MHC variants. There are several genetic areas of great biomedical interest that can provide valuable information on the health and disease predispositions of this population.

An increase in serum ALT levels indicates definite liver cell injury due to a variety of causes. An ALT blood test is often included in a liver panel and in a comprehensive metabolic panel to assess liver damage (Chinchilla-López et al. 2018). To test this, the frequency of the PNPLA3 I148M risk allele was evaluated in indigenous and admixed individuals from Mexico, with the aim of examining its association with liver disease risk. 16 Comcáac individuals were included in this analysis. The results showed that Mexican indigenous populations had the highest frequency of the PNPLA3 148 M risk allele reported to date. This frequency remained similar among the five indigenous groups studied, with the Comcáac community showing the lowest frequency. The association of I148M PNPLA3 with elevated ALT levels was highly significant in both the indigenous and admixed populations (Larrieta-Carrasco et al. 2014).

Methylenetetrahydrofolate reductase (MTHFR), an enzyme crucial for folate metabolism (Leclerc et al. 2013), was also genotyped in the Comcáac population and in 31 other indigenous groups. The C677T polymorphism involves the substitution of an alanine for a valine (A222V), whereas the A1298C variant replaces a glutamic acid with an alanine (E429A). Both polymorphisms have been associated with a decrease in enzyme activity, especially C677T, which confers thermolability to the enzyme. In the study the Comcáac, Pame and Huave populations had lower 677 T allele frequencies than other groups that cohabited the same regions (Contreras-Cubas et al. 2016). Further research is needed to assess the biological relevance of this variant in native populations.

The AKT1, GCKR, and SOCS3 genes have been associated with metabolic syndrome (MS), a major risk factor for cardiovascular disease and T2DM (Devaney et al. 2010; Mohás et al. 2010; Bajpai et al. 2017). This study analyzed eight single nucleotide polymorphisms (SNPs) in these genes in 1923 Native Mexicans from 57 populations and 855 admixed populations. Allele frequencies, genetic associations with metabolic traits, and differences between the two populations were compared. Allele frequencies for rs1130214, rs10141867, rs33925946, rs1260326, rs780094, rs7221341, rs4969168, and rs9914220 variants were reported in 19 Comcáac individuals. The results revealed differences in genetic architecture and differential genetic associations with metabolic traits between Mexican indigenous and Admixed individuals populations, with no particular analysis for our community of interest (Cid-Soto et al. 2018).

A study focused on genes of metabolic interest investigated the association between vitamin D binding protein (VDBP) haplotypes, two CG gene variants (rs7041 and rs4588) and their relationship with vitamin D deficiency (VDD) in postmenopausal women. The study included two groups: Admixed women and participants from 16 Native Mexican populations, including eight Comcáac. Results showed that the Comcáac women had a 70% frequency for the rs7041-G (and 30% T) genotype, and a 10% frequency for the rs4588A (and 90% C) genotype. The study demonstrated that VDD may be associated with GC variants in Mexican-admixed postmenopausal women, but no significant association was found between VDD status and GC polymorphisms in Mexican indigenous postmenopausal women. However, further studies are needed to confirm these findings and determine the impact of these variants on the health of Comcáac women and women from other indigenous communities (Rivera-Paredez et al. 2020).

Obesity in the Comcáac population appears to be shaped by lifestyle change and genetic architecture. In a genome-wide survey of European BMI-associated loci in Mexican children, Costa-Urrutia et al. (2020) analyzed 41 Comcáac girls and 27 boys, finding obesity prevalence of 17.1% and 25.9%, respectively. None of the 15 variants previously linked to childhood BMI in Europeans, several of which were robustly associated with BMI in Central Mexican Mestizo and Nahua children, showed significant association in the Comcáac, underscoring the limited transferability of non-Native genetic predictors and the need for population-specific studies. Differences in mean BMI and obesity prevalence among Native groups have been attributed partly to varying degrees of lifestyle westernization (Abondio et al. 2021), but may also reflect as-yet-undiscovered, population-specific variants. One such variant is the Native American-specific missense mutation R230C in ABCA1, reported by (Acuña-Alonzo et al. 2010) and present in the Comcaac. This allele reduces cholesterol efflux by ~ 27%, lowers HDL-cholesterol, and is associated in other Native and Mestizo populations with higher BMI, obesity, and type 2 diabetes risk; its haplotype structure shows signs of strong positive selection, plausibly as a “thrifty” adaptation to ancestral food scarcity. Hünemeier et al. (2012) linked the R230C frequency pattern in Mesoamerica to maize-based agriculture, yet its presence in the non-agricultural Comcáac suggests a more ancient, widespread dispersal in the Americas. Together, these findings indicate that Comcáac obesity risk is not adequately captured by European-derived loci, and may instead be driven by Indigenous-specific alleles such as ABCA1 R230C, alleles whose historical adaptive benefits may now predispose carriers to dyslipidemia and obesity in a rapidly modernizing socio-dietary environment.

## Conclusions and perspectives

**Fig. 3:**
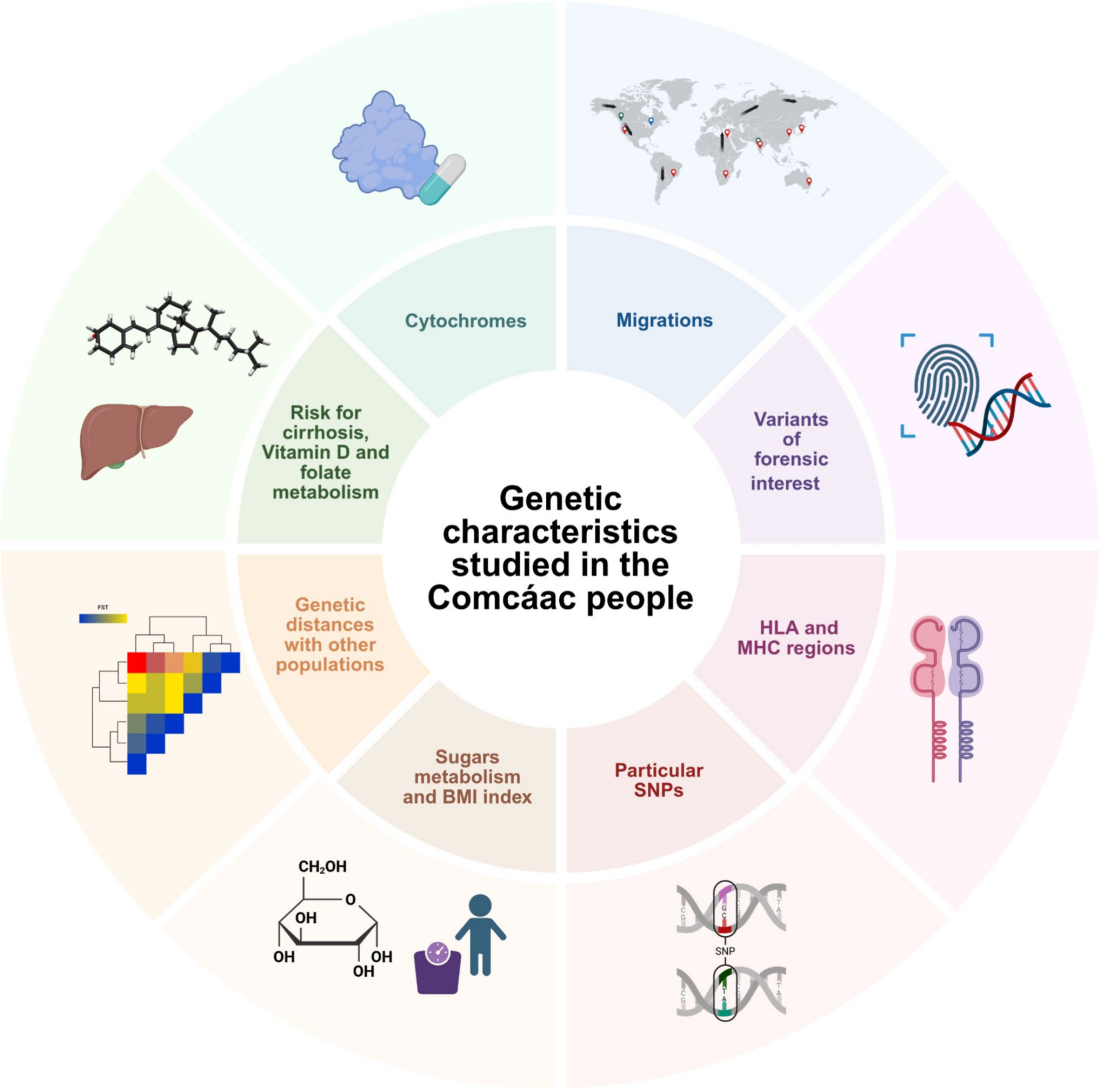
Cytochrome genes, migration patterns, forensic interest variants, HLA and MHC regions, specific SNPs, sugar metabolism and BMI index, genetic distances with other populations, risks associated with cirrhosis, Vitamin D, and folate metabolism, among others have been analyzed in the Comcáac community. Created in Biorender.com

To date, approximately 652 biological samples from the Comcáac population have been analyzed using molecular and omics techniques in at least 36 published studies. These investigations provide insights into variants of forensic interest, immune-related genetic regions, sugar metabolism, BMI index analysis, cirrhosis risk, vitamin D and folate metabolism, cytochromes, and migration patterns (See Fig. 3, Supplementary Table 1). In many of the studies shown here, the Comcáac are reported as a genetically divergent population from other Native Mexican populations, not only through anthropological records and traditional molecular techniques, but also through microarray and omics technologies. The unique genetic variants, high genetic divergence, and elevated homozygosity observed in the Comcáac population could be explained by a bottleneck effect post-European contact. The genetic makeup of the Comcáac appears to have been influenced by geographic barriers and historical demographic events. Factors such as an abrupt population decline, geographic isolation, and a complex geopolitical history may have contributed to their distinctive genetic characteristics. Despite this, there are reports that detect the introduction of genetic elements from European populations (Rey et al. 2012; Sosa-Macías et al. 2013).

Despite these efforts, the molecular landscape of the Comcáac people remains largely uncharted. Notably, there is currently no available data on exome sequencing, gene expression, metabolomics, proteomics, metagenomics, microbiome or single cell omics for this population. Comprehensive genomic and multi-omic investigations have the potential to significantly advance our understanding of population-specific health disparities, ultimately contributing to improved quality of life. Although genetic research has been conducted on the Comcáac community, it has not yet translated into tangible and explicit benefits for its members. A study that integrates genetics into the community’s medical practice has not yet been implemented. There are several actionable points that can be derived from the collected and analyzed data, which could be returned to the community, always considering the clarity and veracity of the true scope and limitations of the information and its interpretation. For example, knowledge about drug-metabolizing cytochromes, if properly communicated and monitored, could positively impact the health of the Comcáac people.

There are promising examples in other human populations that could inspire the scientific community to continue this research. For example, a collaborative study on the high prevalence of sudden cardiac death in the Gitxsan First Nation identified a genetic variant that increases susceptibility to arrhythmia and sudden death (Arbour et al. 2008). Another example is that in New Zealand, research conducted in conjunction with the Ng ¯ati Porou Maori tribe and their health provider, Ng ¯ati Porou Hauora Charitable Trust, discovered genetic variants associated with gout, confirming its hereditary basis. This knowledge has had a significant impact on the community, not only improving diagnosis and treatment of the disease, but also promoting positive lifestyle changes (Tanner et al. 2017).

National and international studies on the genomics of Indigenous populations share common limitations, such as small sample sizes, which hinder the generalization of results to entire populations. Another limitation is the use of tools that are not specifically designed for Indigenous populations, such as commercial arrays. These instruments, optimized for non-Indigenous populations, may introduce biases as they often fail to capture the full genetic diversity of Native American populations. This limitation can impede accurate genetic inferences. Whole-genome sequencing approaches could help address this issue. It is crucial to consider these limitations when interpreting the results of genomic studies in the Comcáac and other Indigenous populations.

Research with appropriate methodologies would be valuable to better understand the history, diversity, and health of these communities. The proposal of an epidemiological summary of the community could be of importance to integrate knowledge. Projects should be carried out with a sensitive, collaborative, and human-centered approach to foster more inclusive, equitable, and fair science. By incorporating community perspectives and including family histories and local needs into research design, we can create a more inclusive scientific landscape. Methods like familial anthropology interviews and the strategic repatriation of knowledge, returning research findings and cultural information to the Comcáac community in an accessible and meaningful way, are crucial components of this process. The Comcáac community exemplifies the rich tapestry of human diversity, history, customs, livelihoods, and unique genetic makeup.

## Methods

To conduct this review on the genetic and genomic discoveries related to the Comcáac people, we performed a comprehensive literature search across multiple scientific databases, including PubMed, Scopus, Web of Science, and Google Scholar. Our search strategy included keywords such as “Comcáac”, “Comcaa’c”, “Konkaak” “Seri,” “native”, “genomics,”, “genetics”,:“population genetics,” “genomic diversity,” and “Native American genetics”.

We included peer-reviewed articles and book chapters that provided insights into the genetic history, population structure, and biomedical relevance of the Comcáac people. Studies were selected based on their relevance and contribution to understanding the genetic diversity of this Indigenous group. Additionally, we reviewed historical and anthropological sources to contextualize genetic findings within the broader cultural and demographic history of the Comcáac.

Data extraction focused on key genetic markers, methods of analysis (e.g., whole-genome sequencing, mitochondrial DNA studies, SNP arrays), and their implications for evolutionary biology, population structure, and health. Where applicable, we compared findings from the Comcáac to those of other Indigenous populations in the Americas to highlight unique genetic adaptations and demographic patterns.

## Supplementary Information

Below is the link to the electronic supplementary material.

Supplementary file1 (PDF 109 KB)

## Data Availability

No datasets were generated or analysed during the current study.
