# Supplementary material for: Genetic and genomic insights of the Comcáac people
Source: J Community Genet. 2025 Sep 30;16(6):653–68. doi: 10.1007/s12687-025-00829-9 (PMC12569335; doi:10.1007/s12687-025-00829-9)
Supplement: Supplementary file 1 — Supplementary file1 (PDF 109 KB) [file 12687_2025_829_MOESM1_ESM.pdf]

Supplementary Table 1. Genetic markers studied on the Comcáac population.

Article	Year	Number of samples obtained/analysed	Marker type	Sample source
Hector et al., 1996	1996	101	HLA genes	Sample collected in the present study
Infante et al., 1999	1999	100	HLA genes	Sample collected in the present study
Gorodezky et al., 2001	2001	100	HLA genes	Infante et al. 1999
Tokunaga et al., 2001	2001	¿?	HLA genes	Infante et al., 1997
Balladares et al., 2002	2002	32	Autosomal SNPs	Sample collected in the present study
Alaez et al. 2002	2002	55	HLA genes	Sample collected in the present study
Malhi et al., 2003	2003	8	mtDNA	Sample collected in the present study
Vargas-Alarcon et al., 2006	2006	100	HLA genes	Petzl-Erler et al. 1997
Schroeder et al., 2007	2007	100	HLA genes	Infante et al. 1999

Malhi et al., 2008	2008	12	Y-chromosomes, mtDNA	Infante et al. 1999
Arnaiz-Villena et al., 2010	2010	100	HLA genes	Petzl-Erler et al., 1997
Rey et al., 2012	2012	100	HLA genes	Petzl-Erler et al., 1997
Rangel-Villalobos et al., 2013	2013	28	Autosomal STRs	Sample collected in the present study
Sosa-Macías, et al., 2013	2013	19	Autosomal STR and SNPs.	Sample collected in the present study
Zúñiga et al., 2013	2013	¿?	HLA genes	Infante et al., 2007
Larrieta-Carrasco et al., 2014	2014	86	Autosomal SNPs	Sample collected in the present study
Moreno-Estrada et al., 2014	2014	24	Genome-wide SNP arrays	Sample collected in the present study
Lazalde-Ramos et al., 2014	2014	19	Autosomal SNPs	Sample collected in the present study
Verdu et al., 2014	2014	3	Genome-wide SNP arrays	Sample collected in the present study

McCulloh et al., 2016	2016	29	Autosomal SNPs	Sample collected in the present study
Contreras-Cubas et al., 2016	2016	19	Autosomal SNPs	Sample collected in the present study
Aguilar-Velázquez et al., 2017	2017	28	Autosomal STRs	Sample collected in the present study
De Andrés et al., 2017	2017	10	Autosomal SNPs	Sample collected in the present study
Ojeda-Granados et al.,2017	2017	19	Genome-wide SNP arrays	Moreno-Estrada et al., 2014
Cid-Soto et al., 2018	2018	19	Autosomal SNPs	Sample collected in the present study
Henderson et al., 2018	2018	38	Autosomal SNPs	De Andrés et al.,2017<br><br>Sosa-Macías, 2013 Lazalde-Ramos, 2014
León-Moreno et al., 2019	2019	19	Autosomal SNPs	Sosa-Macías et al., 2013
Martínez-Cortés et al., 2019	2019	29	Autosomal STRs	McCulloh et al., 2016
Salas-Martínez et al.,2019	2019	19	Autosomal SNPs	Contreras-Cubas., 2016

Costa-Urrutia et al., 2020	2020	68	Autosomal SNPs	Sample collected in the present study
Rivera-Paredez et al., 2020	2020	8	Autosomal SNPs	Sample collected in the present study
Barquera et al., 2020	2020	100	HLA genes	Infante et al. 1999
Aguilar-Ordoñez et al., 2021	2021	4	WGS	Sample collected in the present study
Miron-Toruno et al. 2025	2025	4	WGS	Aguilar-Ordoñez et al., 2021
